# Association between Polymorphisms in Interleukins 4 and 13 Genes and Chronic Periodontitis in a Han Chinese Population

**DOI:** 10.1155/2016/8389020

**Published:** 2016-04-18

**Authors:** Dong Chen, Tian-liang Zhang, Xia Wang

**Affiliations:** ^1^Department of Periodontology, Shanghai Stomatological Disease Center, Shanghai 200001, China; ^2^Medicine Research Center, Weifang Medical University, Weifang 261053, China; ^3^School of Public Health, Weifang Medical University, Weifang 261053, China

## Abstract

Chronic periodontitis (CP) is one of the most common chronic inflammatory diseases and cytokines play a pivotal role in the regulation of immune response. Interleukin-4 (IL-4) and interleukin-13 (IL-13) are anti-inflammatory cytokines and several polymorphisms of them have been proved involved in periodontal disease. This study aimed to evaluate whether three single nucleotide polymorphisms (SNPs), rs2070874 and rs2243248 from* IL4* and rs1800925 from* IL13*, are associated with CP in a Han Chinese population consisting of 440 moderate or severe CP patients and 324 healthy controls. Genomic DNA extracted from buccal epithelial cells of the included participants were genotyped using a matrix-assisted laser desorption/ionization time-of-flight (MALDI-TOF) mass spectrometry method. No significant association between rs2070874 or rs1800925 and CP was found, while the frequencies of rs2243248 and two haplotypes C-G-T and C-T-T showed significant differences between the two groups. The results suggest that the polymorphism rs2243248 and haplotypes C-G-T and C-T-T may be associated with CP susceptibility in the present Han Chinese population.

## 1. Introduction

As a major cause of tooth loss in adults and one of the most common chronic inflammatory diseases, CP is initiated by dental microbial plaque accumulation and characterized by progressive destruction of teeth supporting structures [1]. Although it has been well accepted that CP is initiated by bacterial flora, the dental microbial factor is necessary but not sufficient for the disease onset. Many epidemiological studies have revealed that individuals are often not equally susceptible to the disease [2]. It has been strongly suggested that genes play a vital role in the predisposition to periodontal diseases [3]. Studies of humans and animals indicated that genetic factors could influence inflammatory and immune responses in periodontitis [4–7]. Since it has been shown that attachment loss in periodontitis is induced by Gram-negative bacteria and mediated by inflammatory activation, candidate genes have frequently been selected from proinflammatory and regulatory cytokines which play an important part in immune response regulations [4]. Cytokines, soluble proteins secreted by cells, could transmit signals to other cells as a messenger. They initiate, mediate, and control immune and inflammatory responses [5].

Inflammatory diseases can be triggered and maintained by excessive proinflammatory cytokines or insufficient appropriate anti-inflammatory cytokines production, which seems to be the case in periodontal diseases [8]. Gingival epithelial cells can produce various cytokines including the anti-inflammatory ones such as IL-4 and IL-13 [9–11]. The human* IL4* gene is located within a cluster consisting of other cytokine genes including* IL13* [12]. Several studies have revealed the role of IL-4 in downregulating macrophage function [13–15]. The secretion of prostaglandin E2 (PG-E2) and cytokines by macrophages can be suppressed by IL-4 [14]. Moreover, IL-4 is found to induce monocytes to apoptosis [16]. A localized deficiency of IL-4 might fortify the predisposition to periodontitis [15]. IL-13, another cytokine in common with IL-4, is capable of suppressing the production of proinflammatory cytokines by monocytes/macrophages.

It has been suggested that the susceptibility to CP is related to heritability in approximately 50% of cases [17]. Studies showed that genetic polymorphisms in cytokines could influence their expression and appeared related to the progression of periodontitis [18–20]. Therefore, genetic polymorphisms in the* IL4* gene may aggravate periodontal disease severity by altering IL-4 levels and studies suggested that the -33 C/T^*∗*^T allele was correlated with increased IL-4 expression [21, 22].* IL4* and* IL13* are functionally relevant and only through the combined analyses of genetic polymorphisms in* IL4 *and* IL13* genes could their actual roles in the development of periodontitis be revealed [23]. A number of studies have investigated the correlation between* IL4* -33 C/T (rs2070874) and periodontitis susceptibility [24–27]. Rs2243248, another SNP located in* IL4*, has been found associated with asthma, another chronic inflammatory disease, in both African American and Caucasian children [28]. And several studies have discovered the role of rs1800925 (-1112 C>T) of* IL13* in chronic inflammatory diseases including psoriatic arthritis [29], asthma [30], and rhinitis [31].

SNPs are valuable tools to assess risk allele for a disease since they represent variations in a population. Moreover, those in promoter regions may influence gene expression. Thus, it will be of great interest to investigate whether these three SNPs located in the aforementioned promoter regions (rs2070874, rs2243248, and rs1800925) contribute to the CP susceptibility. To our knowledge, no studies have examined the three SNPs in the Han Chinese population in Shanghai. This report aimed to investigate their association with CP in Chinese individuals.

## 2. Materials and Methods

### 2.1. Study Population and Clinical Assessments

Subjects were recruited from the patients' database of the Department of Periodontology, Shanghai Stomatological Disease Center, ranging from December 2009 to December 2011. Four hundred and forty unrelated patients with moderate or severe CP and 324 ethnically matched controls were included by random selection. The experiment was conducted with the human subjects' understanding and consent after learning the relevant information regarding the study. The demographic information was shown in Table 1. The study was conducted in accordance with the Declaration of Helsinki (1964), and the protocol was approved by the Ethics Committee of Shanghai Stomatological Disease Center (Project identification code: 2009-005).

The CP patients enrolled in the present study should meet one of the following criteria: (1) at least 10 teeth absent, namely, not more than 18 teeth existing, or (2) at least 2 interproximal sites (on different tooth) with clinical attachment loss (CAL) ≥ 6 mm and at least 1 interproximal site with pocket depth (PD) ≥ 5 mm or (3) at least 2 interproximal sites (on different tooth) with CAL ≥ 4 mm or at least 2 interproximal sites (on different tooth) with PD ≥ 5 mm. In addition, the percentage of PD ≥ 4 mm should be in the upper tertile of the study population. These criteria were modified from the definitions proposed by a CDC/AAP working group. The healthy control participants included in the present study did not suffer from any periodontal disease. Exclusion criteria included (1) any past or current systemic disease; (2) any past or current oral disease other than periodontitis; (3) pregnant or lactating females; (4) periodontal or antibiotics treatment in the preceding six months. Moreover, the included subjects were nonsmokers who never smoked, whether in a smoking or smokeless form.

The clinical periodontal parameters including plaque index (PI) and gingival index (GI) for all the participants were also measured as previously described [32]. PI and GI were measured for all teeth except the third molars before periodontal probing. PI and GI scores were calculated by dividing the total score by the number of surfaces examined. The criteria for PI were as follows: score 0 means no plaque on the tooth surface, score 1 means no plaque observed by the naked eye, score 2 means thin plaque visible to the naked eye, and score 3 means abundance of soft matter in the gingival pocket. The criteria for GI were as follows: score 0 means normal gingiva, score 1 means mild inflammation, slight change in color, slight oedema, and no bleeding on probing, score 2 means moderate inflammation, redness, oedema and glazing, and bleeding on probing, and score 3 means severe inflammation, marked redness and oedema, ulceration, and tendency to spontaneous bleeding.

### 2.2. Buccal Epithelial Cells Collection and DNA Extraction

The sampling of the participants' buccal epithelial cells, used for subsequent genomic DNA extraction and SNP genotyping, was performed as previously described [33]. Briefly, the individuals were guided to brush their teeth or rinse the mouth with water at least 2 h prior to saliva collection. 2 mL of saliva was collected using an Oragene® DNA Self-Collection Kit (DNA Genotek, Ottawa, Canada) with preserving fluid inside in advance and conserved at 4°C after being mixed gently with the preserving fluid. And the genomic DNA extraction of samples was performed within the day.

The buccal epithelial cells were gathered by centrifugation at 2000 g for 10 min and the supernatant was discarded. The genomic DNA was extracted according to the classic phenol/chloroform and salt/ethanol precipitation protocol and quantified based on the absorbance at 260 nm by spectrophotometer (General Electric Inc., Fairfield, CT, USA). It was then dissolved in TE buffer (10 mM tris (pH 7.8), 1 mM EDTA) and stored at −70°C until use.

### 2.3. SNP Genotyping

The primers used in the present study for PCR and genotyping were shown in Table 2. The genotyping of the three selected SNPs was conducted by a MALDI-TOF MS method as described previously [34]. The SNPs genotyping was performed through a MassARRAY system (Sequenom, San Diego, California, USA). The genotyping reactions were accomplished using 384-well spectroCHIP (Sequenom, San Diego, California, USA) equipped with a MassARRAY Nanodispenser. The genotype calling was carried out via MassARRAY RT software (version 3.1, Sequenom, San Diego, California, USA) and the generated data was then processed by MassARRAY Typer software (version 4.0, Sequenom, San Diego, California, USA). For quality control, ten percent of the extracted genomic DNA was sampled randomly and genotyped twice and the subsequent genotyping data demonstrated no discrepancies. In addition, all the genomic DNA samples used for genotyping were coded randomly in order to avoid bias.

### 2.4. Statistical Analysis

Quantitative parameters were presented in the form of mean ± standard deviation (SD) unless otherwise indicated. Hardy-Weinberg equilibrium analyses of the SNPs genotypes were performed by chi-square test through comparing the observed genotypes frequencies with the expected ones. To measure the association between CP susceptibility and SNPs alleles or genotypes, contingency table analysis and chi-square test were carried out for odds ratio (OR) and 95% confidence intervals (CI) calculation. And the effects of age and gender were adjusted by means of logistic regression.

The extent of linkage disequilibrium (LD) was measured between each pair of SNPs. The LD, measured by the two most common LD coefficients *D*′ and *r* ^2^ using expectation-maximization algorithm, and haplotype analyses were performed through software named SHEsis [35]. *D*′ = 1 indicates complete LD and *D*′ = 0 demonstrates no LD. Pairwise *D*′ > 0.85 and *r* ^2^ > 0.33 were considered to indicate strong LD [36]. It is considered to be statistically significant if the *P* value is less than 0.05. All statistical analyses were performed using SPSS software (version 19.0, IBM, Chicago, IL, USA).

## 3. Results and Discussion

### 3.1. Results

The genotype frequency distributions of the three SNPs studied in the present work were in accordance with the Hardy-Weinberg equilibrium (Table 3).  Table 4 summarized details of the *P* values and odds ratio. Among these SNPs, there were significant differences in the genotypes and alleles of rs2243248 between CP patients and healthy controls, even after the adjustment for gender and age. The GG and GT genotypes or the allele G were significantly associated with CP, demonstrating a protective role in CP susceptibility (Table 4). No significant association between rs2070874 or rs1800925 and CP was found (Table 4).

LD analysis demonstrated that the three SNPs were in strong LD, with pairwise *D*′ > 0.85 and *r* ^2^ > 0.33 (Table 5). Therefore, to estimate the association between haplotypes and the risk of CP, haplotype analyses were performed using the SHEsis software. The results of haplotype analysis were shown in Table 6 from which we can learn that two haplotypes consisting of rs2070874, rs2243248, and rs1800925 showed significant differences in frequency distributions between CP patients and healthy controls (with *P* values at 1.34*e* ^−005^ for C-G-T and 0.002 for C-T-T, resp.).

### 3.2. Discussion

The genotypic and allelic distributions of SNPs rs2070874, rs2243248, and rs1800925, located in the promoter regions of both* IL4* and* IL13* genes, were examined in a relatively homogeneous Han Chinese population with and without CP. In this study, no statistically significant difference was found in genotypic or allelic distribution between CP and control groups for rs2070874. It is consistent with the investigation in a Czech population where they concluded that the three polymorphisms in* IL4* including rs2070874 act in a cooperative way to exert effects on CP susceptibility [37]. However, contrasting results regarding the significant associations between alleles and genotypes of rs2070874 and CP were also reported in Macedonian and Brazilian populations [25, 26]. This study showed that the CP patients had lower frequencies of the GG genotype or G allele than healthy controls, revealing that rs2243248 GG genotype, or the G allele, may be a protective factor for the onset of CP in the present Han Chinese population. Similar results were also obtained in the aforementioned Macedonian population. The conflicting results reported in different literature may be derived from different ethnicity, small sample size, or various environmental effects on CP. It is possible that one conclusion drawn in one population or racial group may be totally divergent in another. Since racial differences are fairly common in polymorphic systems [38], various alleles or genotypes or genes interacting with one another may exert their influences on the manifestation of the clinical phenotype [39] in different populations. Perhaps this concept could explain the conflicting results regarding the individual polymorphism in different ethnic groups.

No* IL4* mRNA was found in the gingival mononuclear cells freshly separated from patients with advanced stage adult periodontitis and none of the IL-4 protein was detected in their cell culture supernatant, either [40]. And the gingival crevicular fluid (GCF) of CP patients presented the lowest levels of IL-4 compared to healthy controls. Moreover, the plaque index in CP patients displayed a negative correlation with the IL-4 concentration [41]. After settlement of the inflammation, IL-4 was detected in the GCF of patients with severe periodontitis. Therefore, low levels or absence of IL-4 appears to be a trigger for periodontitis [42]. In periodontal tissues, much more CD14, IL1B, TNF-*α*, and E2 were produced by monocytes due to the macrophages accumulation, resulting in bone resorption [15]. The utilization of exogenous recombinant IL-4 in gingival macrophage cultures could cause cell apoptosis, indicating that absence or lower level of IL-4 might enhance the accumulation of macrophages in the diseased periodontium [43]. Therefore, it was suggested that the anti-inflammatory IL-4 exerted effects on periodontitis by suppressing the secretion of proinflammatory cytokines from macrophages [42]. Variations in cytokine levels among patients with periodontitis were documented and associated with periodontitis susceptibility [44]. There are many similarities in the pathogenesis of tissue damage between rheumatoid arthritis (RA) and periodontal disease, implying RA may be a useful model for host response mediated tissue destruction in periodontal disease [45]. In model animals with RA, the tissue destruction was prevented by injecting IL-4 into the lesion compared with the control sites [46]. Those described above illustrate the key role of IL-4 in the pathogenesis of periodontitis, whereas conflicting reports are also available: increased levels of IL-4 were found associated with periodontitis [47, 48]. These differences in observations may be attributed to the ethnic differences in the production efficiency of IL-4.

The expression of IL-13 has also been detected in periodontitis lesions.* IL13* mRNA was detected in 21% of periodontitis samples but in none of the biopsies from healthy controls, suggesting a potential role of* IL13* in the periodontitis onset [49–52]. The C/T exchange at position -1112 leads to the overexpression of IL-13 in Th2 lymphocytes. The -1112 C/T polymorphism was analyzed in patients with aggressive periodontitis in a North European population, demonstrating no significant difference in the genotypic or allelic frequency distributions between the patients and healthy control groups [27]. Regarding the -1112 C/T polymorphism, no significant allelic or genotypic difference in frequency distribution was found in the present work.

Although the individual polymorphism of rs2070874 or rs1800925 alone demonstrated no association with CP in our work, two haplotypes C-G-T (*P* = 1.34*e* ^−005^, OR = 0.415) and C-T-T (*P* = 0.002, OR = 1.867) consisting of rs2070874, rs2243248, and rs1800925 were found significantly correlated with CP (Table 6), implying again the protective role of G allele of rs2243248 to a certain extent. It is likely that each SNP investigated in the present work might exert merely a small effect on functions of the corresponding genes they belong to or on the susceptibility of CP. And as a complex inflammatory disease, the genetic susceptibility of CP is likely to be determined by a number of genes which may interact with each other, while none of them can display a dominating effect on CP susceptibility [53]. And the gene-gene interactions may alter the onset risk of the disease [37]. In addition, deviations may exist in the diagnosis of periodontitis and the standard of clinical examination. And risk factors such as poorly controlled diabetes mellitus, cigarette smoking, osteoporosis, and obesity might also make a difference. We did not investigate these interactions because of the limited data available in the present work. Therefore, in order to confirm the allelic, genotypic, and haplotypic association with susceptibility to or protection against CP, further study employing larger sample size and ethnically diverse populations should be carried out.

Healthy controls enrolled in the present study were slightly younger than CP patients since younger controls may lead to selection bias [54]. And smoking has been considered to be the major environmental risk factor related to increased incidence and severity of periodontitis [55]. It is interesting that sometimes the genetic association with periodontitis susceptibility was obvious only when smokers were excluded [56]. And according to Kornman et al. [57], the risk related to smoking could obscure genetic risk factors. Hence, smokers were excluded in the present study.

Data derived from the present study should be interpreted with caution. First, this study included data only from Han Chinese population; thus, the results can only be extrapolated to this ethnic group. Second, the sample size in the present study is relatively small, though larger than those in many previous studies. It is estimated that thousands of participants are needed to obtain meaningful results for genetic case-control study, since small odds ratios (ranging from 1.1 to 1.50) were obtained in most associations [58]. For further study in the future, in vitro and in vivo functional studies are needed to determine whether the SNP or haplotypes demonstrating significant associations with CP susceptibility in the present work could influence the mRNA or protein levels of* IL4* or* IL13* in the context of CP. In addition, gene-gene and gene-environment interactions and family linkage analysis in different ethnical groups consisting of large-scale samples might be meaningful for obtaining a better insight into the pathogenesis of CP.

## 4. Conclusions

The results suggest that the polymorphism rs2243248 and haplotypes C-G-T and C-T-T may be associated with CP susceptibility in the present Han Chinese population.

## Figures and Tables

**Table 1:** Demographic and clinical characteristics of the study population.

	Healthy controls	CP patients
Age (years; mean ± SD)	50.40 ± 4.60	47.80 ± 5.20
Male (*N*)	195	270
Female (*N*)	129	170
Total	324	440
PI (score; mean ± SD)	1.79 ± 0.26	2.21 ± 0.15
GI (score; mean ± SD)	1.84 ± 0.12	2.35 ± 0.24
PD (mm; mean ± SD)	2.10 ± 0.24	5.90 ± 0.47
CA loss (mm; mean ± SD)	NA	5.20 ± 0.78

Values represent mean ± SD.

NA = not applicable.

**Table 2:** Primers used for genotyping of rs2070874, rs2243248, and rs1800925.

SNPs	Name of primer	Primer sequence
rs2070874	Forward	ACGTTGGATGTGCATCGTTAGCTTCTCCTG
	Reverse	ACGTTGGATGGAGGTGAGACCCATTAATAG
	Single-base extension primer	GCTTCTCCTGATAAACTAATTG

rs2243248	Forward	ACGTTGGATGTGACTAGGAGGGCTGATTTG
	Reverse	ACGTTGGATGGAACCTAAACACATCCTCAG
	Single-base extension primer	GGCTGATTTGTAAGTTGGTAAGAC

rs1800925	Forward	ACGTTGGATGCAACACCCAACAGGCAAATG
	Reverse	ACGTTGGATGAGCCATGTCGCCTTTTCCTG
	Single-base extension primer	TCTGGAGGACTTCTAGGAAAA

**Table 3:** The observed and expected frequencies for genotypes of rs2070874, rs2243248, and rs1800925 testing for the goodness of fit to Hardy-Weinberg equilibrium.

SNPs	Genotype	Observed genotype	Expected genotype	Chi-square	*P* values^*∗*^
rs2070874	Genotype (*N* = 324)				
	T/T	210 (%)	205.4 (%)	2.434	0.119
	C/T	96 (%)	105.2 (%)		
	C/C	18 (%)	13.4 (%)		

rs2243248	Genotype (*N* = 324)				
	T/T	261 (%)	258.7 (%)	1.845	0.174
	G/T	57 (%)	61.6 (%)		
	G/G	6 (%)	3.7 (%)		

rs1800925	Genotype (*N* = 324)				
	C/C	227 (%)	230.9 (%)	2.672	0.102
	C/T	93 (%)	85.3 (%)		
	T/T	4 (%)	7.9 (%)		

^*∗*^Significant difference was accepted at *P* < 0.05.

**Table 4:** Distributions of rs2070874, rs2243248, and rs1800925 polymorphisms in CP patients and healthy controls.

SNP	Genotype/allele	CP patients *N* = 440 (%)	Controls *N* = 324 (%)	Crude OR (95% CI)^†^	*P* value^‡^	Adjusted OR (95% CI)^a^	*P* value^‡^
rs2070874	T/T^*∗*^	289 (65.7)	210 (64.8)	1		1	
	C/T	130 (29.5)	96 (29.6)	0.984 (0.716–1.352)	0.935	1.062 (0.266–4.244)	0.933
	C/C	21 (4.8)	18 (5.6)	0.848 (0.441–1.631)	0.619	1.082 (0.215–5.460)	0.924
	T^*∗*^	708 (80.5)	516 (79.6)	1		1	
	C	172 (19.5)	132 (20.4)	0.950 (0.737–1.224)	0.698	1.216 (0.455–3.247)	0.696

rs2243248	T/T^*∗*^	401 (91.1)	261 (80.6)	1		1	
	G/T	38 (8.6)	57 (17.6)	0.434 (0.280–0.673)	0.001	0.276 (0.237–0.322)	0.002
	G/G	1 (2.3)	6 (1.8)	0.108 (0.013–0.906)	0.018	0.109 (0.085–0.140)	0.001
	T^*∗*^	840 (95.5)	579 (89.4)	1		1	
	G	40 (4.5)	69 (10.6)	0.400 (0.267–0.598)	0.001	0.321 (0.290–0.355)	0.001

rs1800925	C/C^*∗*^	330 (75.0)	227 (70.1)	1		1	
	C/T	96 (21.8)	93 (28.7)	0.710 (0.510–0.989)	0.050	0.826 (0.615–1.109)	0.204
	T/T	14 (3.2)	4 (1.2)	2.408 (0.782–7.408)	0.145	2.091 (1.230–3.556)	0.301
	C^*∗*^	756 (85.9)	547 (84.4)	1		1	
	T	124 (14.1)	101 (15.6)	0.888 (0.668–1.181)	0.422	1.005 (0.736–1.371)	0.977

^a^Adjusted for gender and age.

^†^OR, odds ratio; CI, confidence interval.

^‡^Significant difference was accepted at *P* < 0.05.

^*∗*^Homozygous genotypes were used as reference group.

**Table 5:** Linkage disequilibrium test of rs2070874, rs2243248, and rs1800925.

SNPs	rs2070874	rs2243248	rs1800925
rs2070874	—	0.99	0.99
rs2243248	0.47	—	0.96
rs1800925	0.72	0.60	—

Note: the upper triangle is *D*′ value and the lower triangle is the *r* ^2^ value.

**Table 6:** Haplotype analyses of rs2070874, rs2243248, and rs1800925 for CP patients versus controls.

Haplotype	Case (freq)	Control (freq)	Chi^2^	*P* values^*∗*^	Odds ratio	95% CI^†^
C-G-T	39.99 (0.045)	66.47 (0.103)	18.987	1.34*e* ^−005^	0.415	0.276~0.623
C-T-C	47.99 (0.055)	28.47 (0.044)	0.851	0.356	1.250	0.777~2.011
C-T-T	84.01 (0.095)	34.53 (0.053)	9.151	0.002	1.867	1.239~2.814
T-T-C	708.00 (0.805)	516.00 (0.796)	0.062	0.803	1.033	0.801~1.332

^*∗*^Significant difference was accepted at *P* < 0.05, determined by the Fisher exact test.

^†^CI = confidence interval.
